# Supplementary material for: Closing the pneumococcal conjugate vaccine (PCV) introduction gap: an archetype analysis of last-mile countries
Source: Glob Health Action. 2023 Dec 12;16(1):2281065. doi: 10.1080/16549716.2023.2281065 (PMC10795629; doi:10.1080/16549716.2023.2281065)
Supplement: Supplemental Material [file ZGHA_A_2281065_SM7338.docx]

Table S1 (Supplementary): Data Sources (website links and references) for country selection, landscape desk review and domain-indicator mapping

| Domain | Indicators | Data Source | Website link |
| --- | --- | --- | --- |
| Country Selection | | | |
|  | PCV introduction status | WHO Immunization Data portal | <https://immunizationdata.who.int/> |
|  | Country income status | World Bank Country and Lending Groups | [https://datahelpdesk.worldbank.org/knowledgebase/articles/906519-world-bank-country-and-lending-groups#:~:text=%EF%BB%BF%EF%BB%BF%20For%20the%20current,those%20with%20a%20GNI%20per](https://datahelpdesk.worldbank.org/knowledgebase/articles/906519-world-bank-country-and-lending-groups#:~:text=%25EF%25BB%25BF%25EF%25BB%25BF%2520For%2520the%2520current,those%2520with%2520a%2520GNI%2520per) |
| Mapping of domains and indicators related to vaccine introduction | | | |
| Health characteristics | Incidence rate for pneumococcal diseases (2015) | Burden of Streptococcus pneumoniae and Haemophilus influenzae type b disease in children in the era of conjugate vaccines: global, regional, and national estimates for 2000-15 | <https://www.ncbi.nlm.nih.gov/pmc/articles/PMC6005122/> |
|  | U5 child mortality (U5 child deaths per 1000 live births) | World Bank | <https://data.worldbank.org/indicator/SH.DYN.MORT> |
|  | National health expenditure (% of GDP) | World Bank | <https://data.worldbank.org/indicator/SH.XPD.CHEX.GD.ZS> |
| Immunization systems | Vaccine coverage: DTP3 | World Health Organization- The Global Health Observatory | <https://www.who.int/data/gho/data/indicators/indicator-details/GHO/diphtheria-tetanus-toxoid-and-pertussis-(dtp3)-immunization-coverage-among-1-year-olds-(-)> |
|  | Vaccine coverage: MCV1 | World Health Organization- The Global Health Observatory | <https://www.who.int/data/gho/data/indicators/indicator-details/GHO/measles-containing-vaccine-first-dose-(mcv1)-immunization-coverage-among-1-year-olds-(-)> |
|  | Recent new vaccine introductions (RV, HPV, IPV, MCV2) | WHO Immunization Dashboard Global | <https://immunizationdata.who.int/index.html> |
|  | PCV partial introduction | WHO Immunization Data portal | <https://immunizationdata.who.int/> |
| Policy framework | Presence of a National Immunization Technical Advisory Group (NITAG) | Global NITAG Network (GNN) | [Map \| NITAG RESOURCE CENTER (nitag-resource.org)](https://www.nitag-resource.org/network/map) |
|  | Political will pertaining to PCV introduction | Sourced from the government, in-country health partners, public announcements, published accounts | N/A |
|  | Gavi-eligibility and support | | |
|  | 1. Gavi status | Gavi Programmes and Impact- Country hub | <https://www.gavi.org/programmes-impact/country-hub> |
|  | 1. Gavi Middle-Income Countries Approach | Gavi Types of Support- Making Immunisation Sustainable | <https://www.gavi.org/types-support/sustainability/gavi-mics-approach> |

Table S2 (Supplementary): Validation of scoring framework, countries that introduced PCV in 2021 and 2022

| Country and year of PCV introduction | Pneumococcal incidence rate (2015) | U5 child mortality rates per 1,000 live births | National health expenditure (% of GDP) | Vaccine coverage: DTP3^1^ | Vaccine coverage MCV1^2^ | Recent new vaccine introductions (RV^3^, HPV^4^, IPV^5^, MCV2^6^) | Partial PCV introduction | Presence of a NITAG | Political will pertaining to PCV introduction | Gavi eligibility & support | Score | Country Category based on score |
| --- | --- | --- | --- | --- | --- | --- | --- | --- | --- | --- | --- | --- |
| Tajikistan (2022) | 1 | 1 | 2 | 4 | 4 | 3 | 0 | 3 | 4 | 3 | 25 | Low-barrier |
| India (2021) | 3 | 1 | 1 | 3 | 3 | 3 | 2 | 4 | 4 | 0 | 24 | Low-barrier |
| Timor Leste (2022) | 2 | 2 | 2 | 3 | 3 | 3 | 0 | 3 | 4 | 2 | 24 | Low-barrier |
| Indonesia^a^ (2022) | 3 | 1 | 1 | 2 | 2 | 2 | 2 | 4 | 4 | 2 | 23 | Low-barrier |
| Tuvalu (2021) | 1 | 1 | 4 | 4 | 4 | 4 | 0 | 0 | 4 | 1 | 23 | Low-barrier |
| Samoa (2021) | 2 | 1 | 2 | 3 | 2 | 3 | 0 | 2 | 4 | 1 | 20 | Low-barrier |
| Tonga (2021) | 2 | 1 | 1 | 4 | 4 | 3 | 0 | 0 | 4 | 1 | 20 | Low-barrier |
| Vanuatu (2021) | 2 | 1 | 1 | 3 | 3 | 2 | 0 | 0 | 4 | 1 | 17 | Moderate |

^a^ Indonesia partially introduced PCV in 2017 but it was halted, and later reintroduced in 2022.
